# Comparison of fruit morphology and nutrition metabolism in different cultivars of kiwifruit across developmental stages

**DOI:** 10.7717/peerj.11538

**Published:** 2021-06-23

**Authors:** Yu-fei Li, Weijia Jiang, Chunhong Liu, Yuqi Fu, Ziyuan Wang, Mingyuan Wang, Cun Chen, Li Guo, Qi-guo Zhuang, Zhi-bin Liu

**Affiliations:** 1 Sichuan University, Key Laboratory of Bio-Resources and Eco-Environment of Ministry of Education, College of Life Sciences, Chengdu, China; 2 West China Medical Publishers, West China Hospital of Sichuan University, Chengdu, China; 3 College of Chemistry and Life Sciences, Sichuan Provincial Key Laboratory for Development and Utilization of Characteristic Horticultural Biological Resources, Chengdu Normal University, Chengdu, China; 4 Kiwifruit Breeding and Utilization Key Laboratory, Sichuan Provincial Academy of Natural Resource Sciences, Chengdu, China

**Keywords:** Kiwifruit, Development process, Fruit composition, Carbohydrate metabolism, Organic acid, Antioxidant enzyme system

## Abstract

Kiwifruit (*Actinidia*) is becoming increasingly popular worldwide due to its favorable flavour and high vitamin C content. However, quality parameters vary among cultivars. To determine the differences in quality and metabolic parameters of kiwifruit, we monitored the growth processes of ‘Kuilv’ (*Actinidia arguta*), ‘Hongyang’ (*Actinidia chinensis*) and ‘Hayward’ (*Actinidia deliciosa*). We found that ‘Kuilv’ required the shortest time for fruit development, while ‘Hayward’ needed the longest time to mature. The fruit size of ‘Hayward’ was the largest and that of ‘Kuilv’ was the smallest. Furthermore, ‘Hongyang’ showed a double-S shape of dry matter accumulation, whereas ‘Kuilv’ and ‘Hayward’ showed a linear or single-S shape pattern of dry matter accumulation during development. The three cultivars demonstrated the same trend for total soluble solids accumulation, which did not rise rapidly until 90–120 days after anthesis. However, the accumulation of organic acids and soluble sugars varied among the cultivars. During later fruit development, the content of glucose, fructose and quinic acid in ‘Kuilv’ fruit was far lower than that in ‘Hongyang’ and ‘Hayward’. On the contrary, ‘Kuilv’ had the highest sucrose content among the three cultivars. At maturity, the antioxidative enzymatic systems were significantly different among the three kiwifruit cultivars. ‘Hongyang’ showed higher activities of superoxide dismutase than the other cultivars, while the catalase content of ‘Hayward’ was significantly higher than that of ‘Hongyang’ and ‘Kuilv’. These results provided knowledge that could be implemented for the marketing, handling and post-harvest technologies of the different kiwifruit cultivars.

## Introduction

Kiwifruit (*Actinidia*) has become one of the most popular fruits in recent years. It has a high nutritional value, containing mineral elements, dietary fiber, amino acids, and folate. Meanwhile, it also has abundant antioxidants, enzymes, and phytonutrients (Richardson, Ansell & Drummond, 2018). The dietary fiber effectively facilitates gastrointestinal peristalsis (Stonehouse et al., 2013). Folate reduces the risk of cancer (Choi & Mason, 2000), and a special phytonutrient named actinidin can help digest proteins in the gastric area (Kaur et al., 2010). Moreover, kiwifruit is famous for its high vitamin C (VC) content, which can reach 420 mg 100 g^−1^ in some cultivars, much higher than that in other fruits (Du et al., 2009). VC is an antioxidant that can prevent pathologies such as cancer and cardio-vascular diseases (Tavarini et al., 2008). The high nutritional value, together with the good appearance and taste, has made kiwifruit an important economic fruit crop, and its planting area has increased dramatically all over the world (Tang et al., 2016). The total yield of kiwifruit is increasing year by year globally, with China, Italy, and New Zealand producing the highest yields; the total yield of kiwifruit in these three countries accounts for more than half of the global yield (Ma et al., 2017).

At present, the most widely planted kiwifruit species in the world are *Actinidia deliciosa* and *Actinidia chinensis.* There are many cultivars of *A. deliciosa,* among which ‘Hayward’ is planted most widely, representing around 90% of kiwifruit in the world (Garcia et al., 2012). From a commercial perspective, fruits of ‘Hayward’ are appealing to customers because of their bright translucent green flesh and satisfactory storage ability. Furthermore, ‘Hayward’ is planted in many countries due to its high productivity, greater weight, and higher content of soluble solids compared with other cultivars (Burdon et al., 2004). Besides *A. deliciosa*, some *A. chinensis* cultivars also have high commercial value and are planted worldwide. ‘Hongyang’ is a smooth-skinned and almost hairless *A. chinensis* cultivar. It is the first commercial red-flesh variety (Ma et al., 2017) and has a relatively higher sugar/acid ratio than other cultivars, producing better flavour, which is popular among customers (Nishiyama et al., 2008). Compared with ‘Hayward’, ‘Hongyang’ has higher VC content (Wang, Li & Meng, 2003); however, is fruits are relatively smaller than those of ‘Hayward’ and plants are more sensitive to bacterial canker, so fruit price is higher than that of ‘Hayward’(Costa et al., 2018). *Actinidia argute* has gained great importance in the agricultural product industry recently, with ‘Kuilv’ representing a typical cultivar. ‘Kuilv’ fruits are smaller than traditional kiwifruit such as ‘Hayward’ but have a superb flavour and hairless, edible skin, making them convenient to eat (Williams et al., 2003). Moreover, in terms of cultivation, ‘Kuilv’ can withstand multiple stresses, such as low temperatures of up to −30 °C and pests and diseases, which can reduce the planting costs (Drzewiecki et al., 2016).

**A**biotic and biotic stresses cause the accumulation of reactive oxygen species, such as O^2−^, H_2_O_2_, singlet oxygen, and hydroxyl radicals; excessive accumulation of reactive oxygen species can initiate and accelerate a membrane lipid peroxidation chain reaction, thereby producing large amounts of harmful substances such as Malondialdehyde (MDA), which can further poison cells (Wang et al., 2020). However, fruits and vegetables can protect themselves naturally against oxidative damage by producing antioxidant enzymes, such as Superoxide Dismutase (SOD), Catalase (CAT), and Peroxidase (POD). The activity of antioxidant enzymes in different kiwifruit cultivars has not yet been compared.

The sugar-acid ratio may influence fruit taste and flavour and is an important parameter for the classification of kiwifruit cultivars (Zaouay et al., 2012). Previous research has concentrated on the sugar and organic acid content of mature kiwifruit, but there have been few studies on the variations in sugar and organic acid content in different cultivars during growth.

The development of fruit from anthesis to ripening has been widely reported for ‘Hayward’ and ‘Hongyang’ (Zhang et al., 2018; Ferrandino & Guidoni, 1998; Nardozza et al., 2017; Ainalidou et al., 2016). These cultivars show significant differences in development, such as in growth rate and fresh weight. However, these studies were performed in different regions at different times, making it hard to conclude the growth differences between cultivars. Moreover, as a new kiwifruit cultivar, the physiological processes at each developmental stage of ‘Kuilv’ have been poorly studied.

Therefore, in this study, we set out to compare the growth and nutrition metabolism of ‘Hayward’, ‘Hongyang’ and ‘Kuilv’ in order to reveal the origins of the differences among the different kiwifruit cultivars.

## Materials & Methods

### Experimental materials and field experiments

The kiwifruit cultivars used in this experiment were ‘Kuilv’, ‘Hongyang’ and ‘Hayward’. Field experiments were carried out in Mianyang of Sichuan Province (31°13′N, 104°16′E) in 2018. The vines were trained to a Pergola system with row-to-row distance of 4 m and plant-to-plant distance of 3 m. Ten kiwi fruit vines of the same age (5-years-old), showing good growth trends and orientation and the same fruit load, were selected for each cultivar. All field experiments were performed as typical for commercial kiwifruit orchards in the area, with water supplied by drip irrigation. At 15 days after anthesis (DAA), fruits of each cultivar were selected randomly from different parts of the vine, marked for subsequent experiments and harvested when appropriate. They were first picked at 30 DAA, followed by harvests at 15-day intervals (10 fruits per vine). The samples were placed in separate paper storage bags and marked. Fruit samples were collected in four biological replicates, snap-frozen in liquid nitrogen, and then stored at −20 °C for subsequent analysis. When measuring physiological indexes, the samples were pressed into a pulp using a juicer at low temperature, in order to reduce any unnecessary chemical reactions.

### Evaluation of fruit weight and shape

At regular intervals (15 days apart), four fruits were picked from 10 labelled kiwifruit vines for each kiwifruit cultivar. Fruit fresh weight was measured according to the method reported by Nardozza et al. (2010). Shape factors such as length and the minimum and maximum diameters were measured using a Vernier caliper.

### Measurement of total soluble solids (TSS), dry matter (DM), starch and total soluble sugars and carbohydrates

To determine the TSS, juice was obtained from both ends of the fruit and its content of TSS was determined using a refractometer (WYT-4, China). The DM content was measured after drying a 2-mm-thick cross-sectional slice per fruit sample for 24 h at 65 °C, according to the method reported by Nardozza et al. (2010).

Starch assay was performed by colorimetry after samples were digested enzymatically, as described by Qiu et al. (2020) with the following modifications. After centrifugation with 70% ethanol, the supernatant was used to determine soluble sugars and the pellet was used to assay starch accumulation. Anthranone sulfate solution was heated at 100 °C for 11 min and cooled on ice. Absorbance was read at 630 nm. The pellet was dissolved using 1.1% HCl and boiled for 30 min. After cooling and centrifugation, the supernatant was collected. The subsequent procedure was the same as for the measurement of soluble sugars.

Carbohydrate accumulation was measured and calculated using the method described by Barboni & Chiaramonti (2006), using an Agilent 1200 Series instrument with a refractive index detector (Agilent, USA). After that, the extract was separated using an amino column (Inertsil NH_2_ 250 mm × 4.6 mm, 5 µm), and then eluted isocratically for 20 min at 85 °C.

### Titratable acid (TA)

TA was determined by the traditional titratable method described by Suh, Jung & Lee (2018). Briefly, 20 mL of the juice was titrated with 0.1 M NaOH. Organic acids were assessed according to the method of Zheng et al. (2009). Briefly, 10 g of kiwifruit was transferred into a 50 mL centrifuge tube containing 12.5 mL NH_4_H_2_PO_4_ (40 mmol/L, pH 2.5) and centrifuged at 12,000× g at 4 °C for 15 min. The supernatants were collected after being filtered (0.45 µm filter, Millipore, Bedford, USA) and stored at 4 °C for further analysis. HPLC analysis was performed using an Agilent 1200 Series device with a C18 column (4.6 mm ×250 mm, 5 µm; Waters, UK) at a wavelength of 210 nm. The mobile phase was NH_4_H_2_PO_4_ (40 mmol/L, pH 2.5).

### Evaluation of VC content

VC content was measured using the 2, 6-dichlorophenol titration method (Alhassan et al., 2019). Briefly, 20 mL of 1% oxalic acid solution was added to 5 g of thawed kiwi fruit pulp. The solution was shaken, and the volume of the solution was adjusted to 50 mL. Samples were placed in the dark for 2 h, centrifuged at 8,000× g at 4 °C for 10 min, and 10 mL of the supernatant was used to determine the VC content. The extract was titrated with 2,6-dichlorophenol indophenol salt solution (1 mL ≈ 0.02 mg VC) until the solution color became light red and remained unchanged for 15 s. The titration was repeated three times, and the volume of 2,6-dichlorophenol indophenol salt solution used for titration was calculated.

### Superoxide dismutase (SOD), peroxidase (POD) and catalase (CAT) activity assays

SOD activity was determined using the method of Xu (2019) (Yang et al., 2015). Briefly, 0.05 mL of enzyme extract was added to sodium phosphate buffer (0.1 M, pH 7.4), followed by addition of 1.35 mL detection reagent consisting of methionine, Ethylene Diamine Tetraacetic Acid (EDTA), Nitrotetrazolium Blue chloride (NBT) and riboflavin. The reaction was maintained in a 37 °C water bath for 30 min. Absorbance at 550 nm was measured using a spectrophotometer (TU-1901, Unico, China), and sodium phosphate buffer (0.1 M, pH 7.4) was used for comparison. One unit of enzyme activity was defined as the amount of enzyme that could inhibit 50% of the reduction of NBT.

POD activity was measured using the method described by Yang et al. (2015) using guaiacol as the donor and H_2_O_2_ as the substrate (Yang et al., 2015). One unit of POD activity was defined as an increase in absorbance of 0.01 per minute at 460 nm under the measurement conditions.

CAT activity was determined according to the method of Xu (2019) with modifications. The test mixture included 2.25 mL of H_2_O_2_ prepared using sodium phosphate buffer (0.1 M, pH 7.4) and 0.05 mL of enzyme solution (Xu et al., 2019). Increase in absorbance at 240 nm was recorded at 25 °C for 3 min. One unit of CAT activity was defined as the amount of enzyme extract that caused a 0.01 min^−1^ change in absorbance.

### Data analysis

Experimental data are presented as the means ± standard errors from three independent experimental replications. Data were analyzed using SPSS version 23.0 (SPSS, Chicago, IL, USA). A single factor ANOVA (ANOVA-LSD) was used to make comparisons among groups. A difference was considered statistically significant at *p* < 0.05.

## Results

### Fruit growth and development of the three species

The flowering period and ripening time were dramatically different among the cultivars, although they were planted in the same resource orchard. The full-bloom period of ‘Kuilv’ and ‘Hongyang’ occurred in the middle of April, one month earlier than that of ‘Hayward’ (the middle of May). Meanwhile, the ripening time of ‘Kuilv’ was in late August, and the total fruit development period was about 120 days, which was shorter than those of ‘Hongyang’ (135 DAA) and ‘Hayward’ (165 DAA).

**Figure 1:**
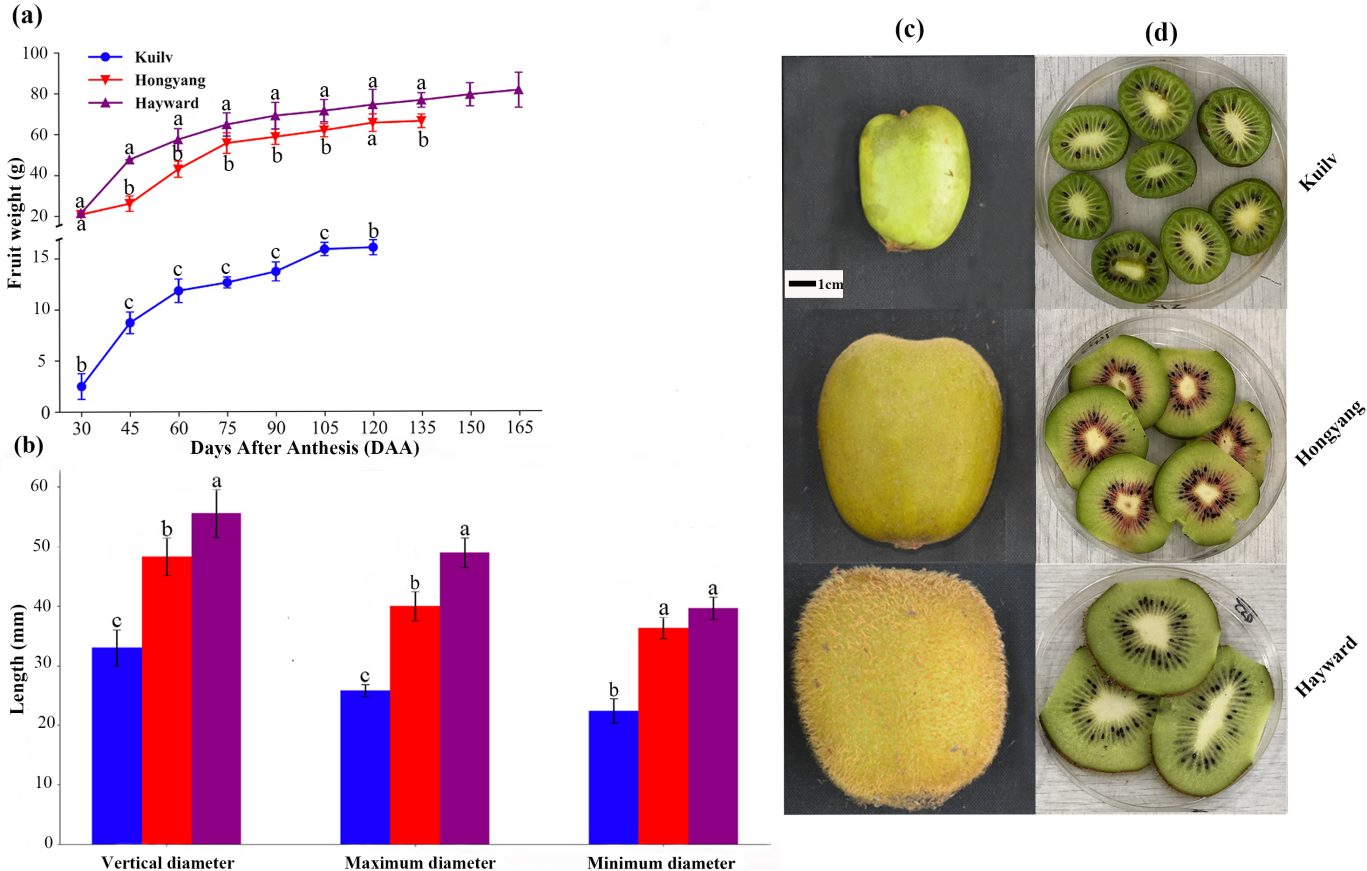
The weight (A), diameter (B), size (C) and flesh (D) of the three cultivars of kiwifruit. DAA, Days After Anthesis. Values are mean ± standard error (SE) from four biological replicates. Different letters indicate significant differences at *P* < 0.05.

Although the fruit weight of the three cultivars increased throughout the growth period, there were still some differences. Throughout the growth period, the fruit weight of ‘Hayward’ and ‘Hongyang’ showed a single ‘S’ shape, which could be divided into three stages: rapid, slow and stagnant growth periods. At the end of the rapid growth period (75 DAA for ‘Hongyang’ and ‘Hayward’), the fresh weight of ‘Hongyang’ and ‘Hayward’ was up to 84.06% and 62.46% of that at fruit harvest. During the stagnant growth period, the fruit weight remained steady, and the final fruit weights of ‘Hongyang’ and ‘Hayward’ were about 66.18 g and 81.29 g, respectively. In comparison, the ‘Kuilv’ fruit weight showed a double-S-shaped growth curve throughout the growth period (Fig. 1A), suggesting that ‘Kuilv’ fruit had two rapid growth periods. In the first rapid growth period, the ‘Kuilv’ fruit weight reached 78.80% of the final fruit weight. In the second rapid growth period following the slow growth period, it reached 98.97% of the final fruit weight. The final harvest weight of ‘Kuilv’ was about 16.04 g, which was far less than those of ‘Hayward’ and ‘Hongyang’.

The vertical diameter, maximum diameter and minimum diameter of fruit are important characteristics indicative of fruit quality. Figure 1B shows the shape and size of fruits of ‘Hayward’, ‘Hongyang’ and ‘Kuilv’ at the time of picking. The ‘Hayward’ fruit had the largest size while the ‘Kuilv’ fruit had the smallest size. The vertical diameter, maximum diameter and minimum diameter were 32.99 mm, 25.81 mm and 22.44 mm for the ‘Kuilv’ fruit; 48.29 mm, 40.00 mm and 36.33 mm for the ‘Hongyang’ fruit, and 55.55 mm, 48.97 mm and 39.16 mm for the ‘Hayward’ fruit (Fig. 1B), respectively.

Figures 1C and 1D shows the size and vertical section of ‘Hayward’, ‘Hongyang’ and ‘Kuilv’ fruits when they were picked. The ‘Kuilv’ fruit had dark green flesh with no hair on the skin. The ‘Hongyang’ fruit had yellow flesh with a red center and a hairless skin. The ‘Hayward’ fruit had green flesh with dense hair on the skin.

### Carbohydrate changes in the three species during fruit development

**Figure 2:**
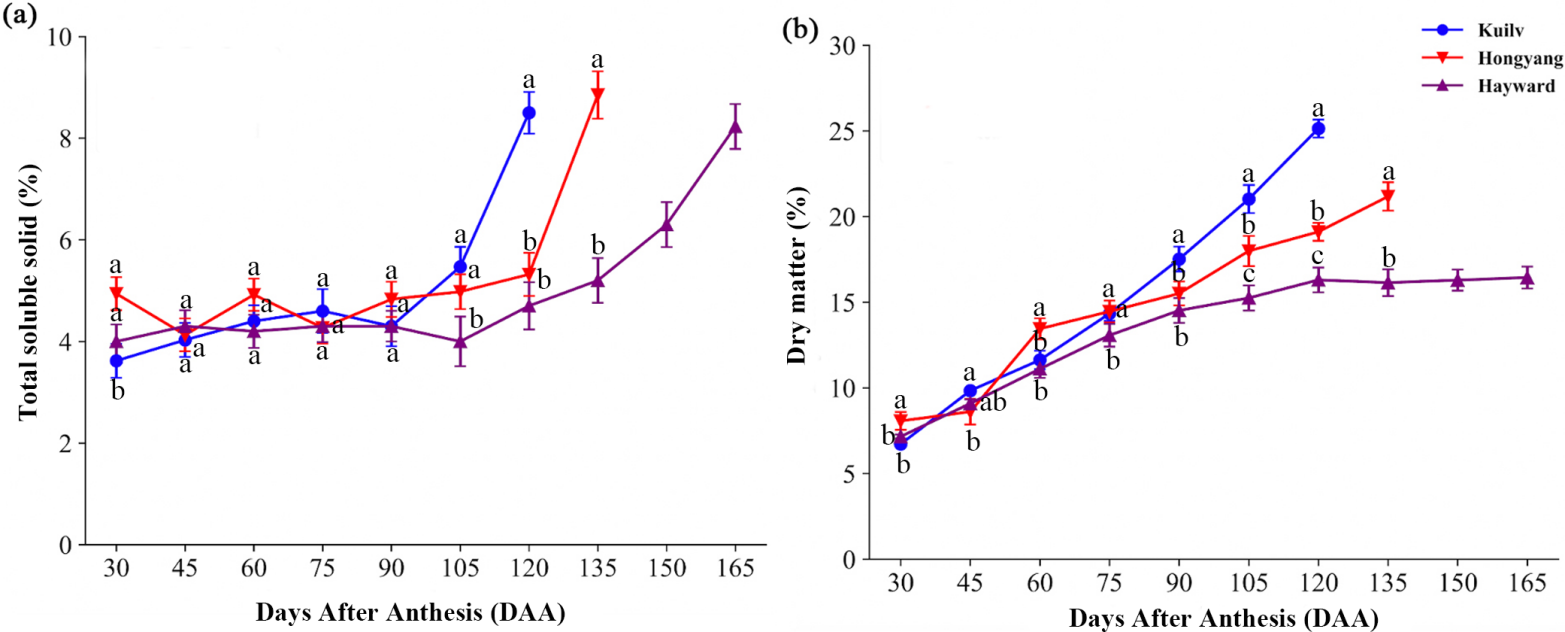
Total soluble solid content (A) and dry matter (B) in three cultivars of kiwifruit. DAA, Days After Anthesis. Values are mean ± standard error (SE) of four biological replicates. Different letters indicate significant differences at *P* < 0.05.

In the early stage of fruit development, there was little fluctuation in TSS content of all species; TSS then increased dramatically before the fruit matured (Fig. 2A). The fruit TSS content in ‘Hayward’ was lower (8.23%) at later stages than that in ‘Hongyang’ and ‘Kuilv’ (around 8.85% and 8.5%, respectively).

Fruit of the three species all showed a trend of increasing DM with developmental age, yet with different accumulation patterns (Fig. 2B). Specifically, the DM of ‘Hayward’ fruit increased linearly from 30 DAA to 120 DAA. This was followed by a second, slower phase of fruit growth from 120 DAA to 165 DAA, during which the DM remained constant (16.29%). In contrast, the DM of ‘Hongyang’ fruit increased throughout development, with a relatively higher accumulation rate at 45–60 DAA and 90–105 DAA. The DM of ‘Kuilv’ fruit increased in a linear pattern, ranging from 6.72% to 25.14%.

**Figure 3:**
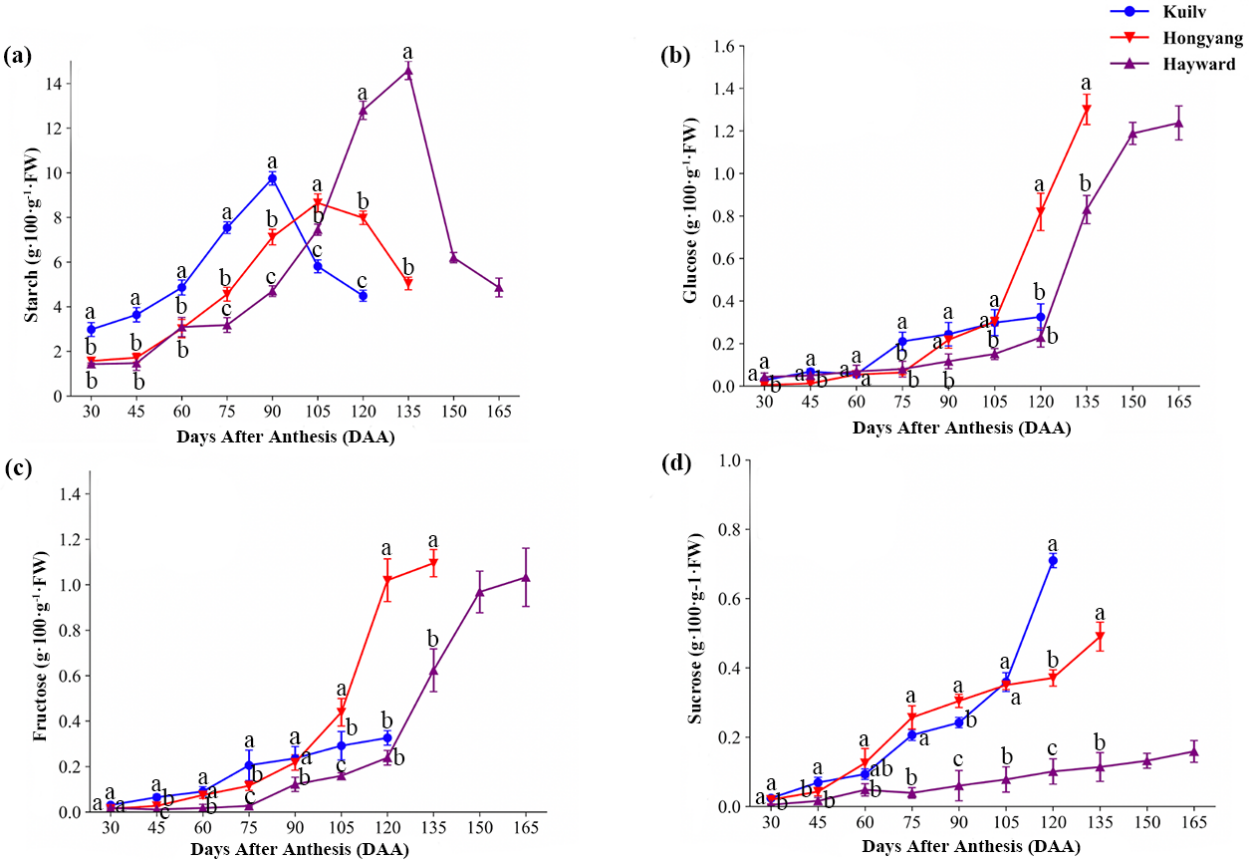
The content of starch (A), glucose (B), fructose (C) and sucrose (D) in the three cultivars of kiwifruit. DAA, Days After Anthesis. Values are mean ± standard error (SE) of four biological replicates. Different letters indicate significant differences at *P* < 0.05.

The three species had similar patterns of starch accumulation during fruit development (Fig. 3A). In ‘Kuilv’, which had the shortest time of fruit growth, the starch concentrations peaked at 90 DAA, earlier than the other species (105 DAA for ‘Hongyang’; 135 DAA for ‘Hayward’), and were lowest at the time of harvest. As the fruit weight continued to increase, starch degradation began to occur, coinciding with a fast rate of sugar accumulation (glucose, fructose and sucrose) in all three species.

Interestingly, the glucose concentration in ‘Kuilv’ fruit did not show a rapid increase before harvest (Fig. 3B). From 75 DAA to 120 DAA, the glucose concentration steadily increased, and ‘Kuilv’ had the lowest glucose concentration among the three cultivars. In contrast, the amount of glucose increased greatly from 105 DAA to 135 DAA in ‘Hongyang’ and from 120 DAA and 150 DAA in ‘Hayward’ fruits. There was a decrease of glucose accumulation in ‘Hayward’ before harvest, and the concentration in ‘Hongyang’ (1.30 g 100 g^−1^ FW) was slightly higher than that in ‘Hayward’ (1.24 g 100 g^−1^ FW).

In addition, fruit of ‘Kuilv’ showed only a small change (from 0.03 g 100 g^−1^ FW to 0.33 g 100 g^−1^ FW) in fructose content, while the fructose concentrations in ‘Hayward’ and ‘Hongyang’ fruits increased rapidly before maturity (Fig. 3C).

The sucrose content increased smoothly within a small range in ‘Hayward’ fruit (Fig. 3D). ‘Hongyang’ showed a similar trend to ‘Hayward’ of stable sucrose content, but a higher rate of increase compared with ‘Hayward’. By contrast, ‘Kuilv’ fruit showed a relatively greater change in sucrose content during development, which increased continuously to 0.71 g 100 g^−1^ FW, higher than those of the other two cultivars, which were 0.16 g 100 g^−1^ FW and 0.49 g 100 g^1^ FW, respectively, at harvest.

### Changes in organic acid content

**Figure 4:**
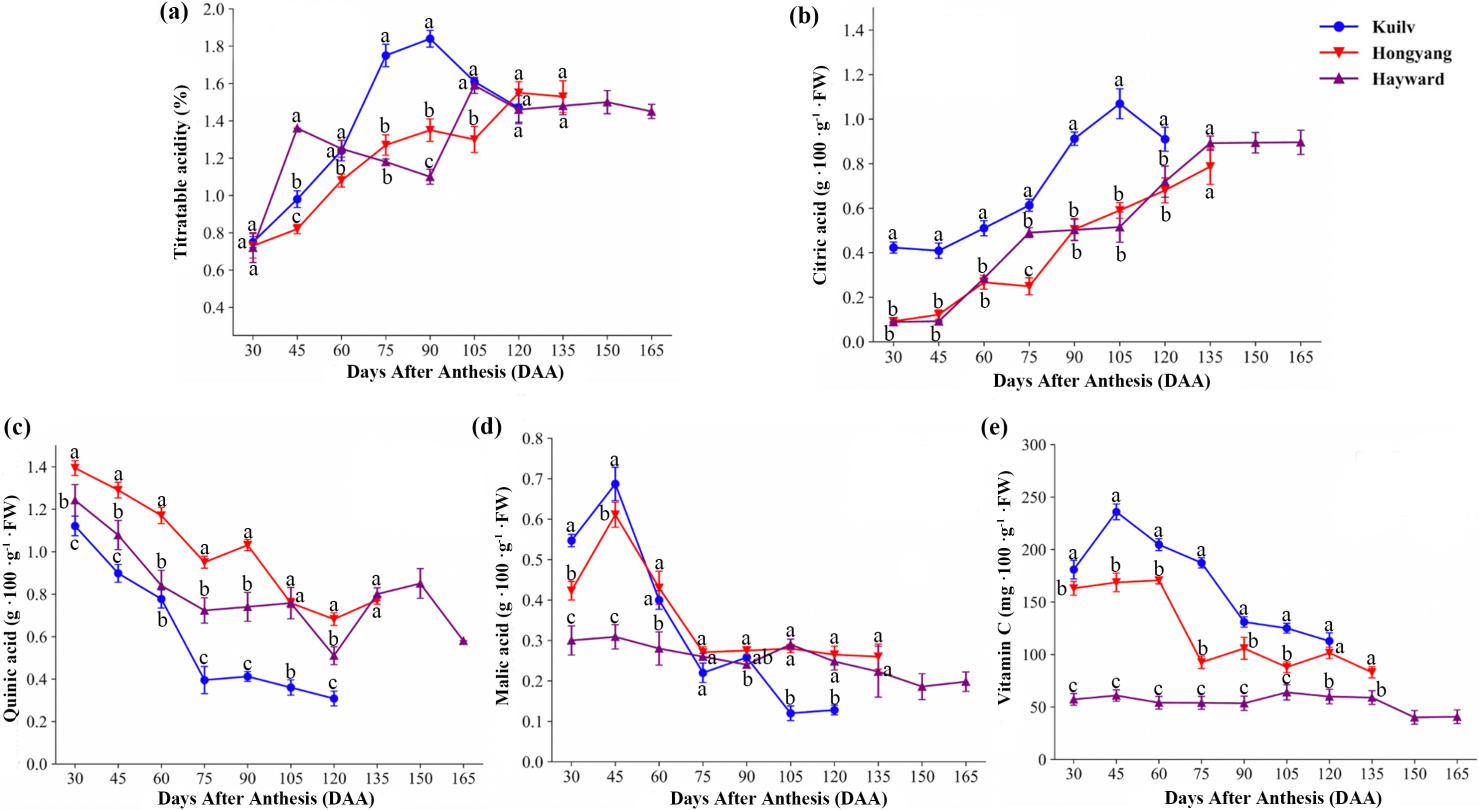
The content of titratable acid (A), citric acid (B), quinic acid (C), malic acid (D) and vitamin C (e) in fruits of the three cultivars of kiwifruit. DAA, Days After Anthesis. Each value indicates the mean ± standard error (SE) of four biological replicates. Different letters indicate significant differences at *P* < 0.05.

We found significant differences in fruit acidity among the three kiwifruit cultivars throughout the growth period (Fig. 4). There was a rapid increase in titratable acid content in ‘Hayward’ fruit from 30 DAA to 45 DAA, reaching a maximum of 1.36% (*w*/*w*), then a decline from 45 DAA to 90 DAA (Fig. 4A). The total titratable acidity of ‘Kuilv’ fruit increased consistently, then decreased from 90 DAA to 120 DAA. However, the titratable acidity of ‘Hongyang’ fruit increased steadily until fruit maturity.

The concentration of citric acid (Fig. 4B) in fruits increased over time in the three species, but was apparently higher in ‘Kuilv’ than in the other two cultivars. Moreover, it decreased from 105 DAA to 120 DAA after reaching a peak (1.069 g 100 g^−1^ FW) at 105 DAA in ‘Kuilv’. Meanwhile, the citric acid continued to accumulate during maturation in fruits of the other two species.

Unlike citric acid, the level of quinic acid (Fig. 4C) eventually declined in all three cultivars, but with different patterns of decrease. The quinic acid levels increased again during fruit maturation in ‘Hayward’ and ‘Hongyang’; for ‘Hayward’, the pick-up time was 120–150 DAA, while that for ‘Hongyang’ was 75–90 DAA. However, ‘Kuilv’ did not show an obvious increase in quinic acid during maturation and had the lowest level of quinic acid (0.31 g 100 g^−1^ FW).

Malic acid (Fig. 4D) also decreased during the fruit development process, but all three cultivars showed an initial increase at 30–45 DAA, followed by a drop until maturity. At harvest, the content of malic acid in ‘Kuilv’ fruit was lower (0.13 g 100 g^−1^ FW) than those in ‘Hongyang’ (0.26 g 100 g^−1^ FW) and ‘Hayward’ (0.20 g 100 g^−1^ FW) fruits.

In addition, the content of VC in ‘Kuilv’ fruit peaked at 45 DAA and then gradually decreased until harvest. The content of VC in ‘Hongyang’ fruit increased to the highest level at 60 DAA, then declined in a wave form, reaching the lowest level of 83.02 mg 100 g^−1^ FW at 135 DAA. The VC concentration of ‘Hayward’ also fluctuated, but within a smaller range. In addition to the differences in the trend of change with time, the VC content also differed among the three kiwi fruits. ‘Kuilv’ had a higher VC content than ‘Hongyang’ or ‘Hayward’ at any time.

### SOD, POD and CAT antioxidant enzyme activities in the three species

**Figure 5:**
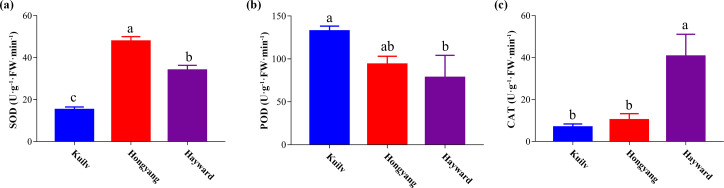
SOD (A), POD (B), and CAT (C) antioxidant enzyme activities profiles of the three cultivars of kiwifruit. Each value indicates the mean ± standard error (SE) of four biological replicates. Different letters indicate significant differences at *P* < 0.05.

After kiwi fruit ripening, the SOD activity of ‘Hongyang’ fruit was highest among the three cultivars, 40.57% and 114.90% greater than that of ‘Hayward’ and ‘Kuilv’ fruit, respectively (Fig. 5A). In contrast, the POD activity of ‘Kuilv’ fruit was 115.94 U g^−1^ FW min^−1^, significantly higher than those of ‘Hayward’ and ‘Kuilv’ fruits (Fig. 5B). The CAT activity of ‘Kuilv’, ‘Hongyang’ and ‘Hayward’ fruits were 6.84 U g^−1^ FW min^−1^, 10.28 U g^−1^ FW min^−1^ and 31.08 U g^−1^ FW min^−1^, respectively (Fig. 5C).

## Discussion

Fruit weight and shape have economic significance because the market price is partially based on these properties. Here we found that the rapid growth in fresh weight of the three types of kiwifruit generally occurred around 30 DAA, although that of ‘Hongyang’ was later than 30 DAA (Kim, Beppu & Kataoka, 2012). The fruit weight did not decrease during the harvesting period. In addition, ‘Hongyang’ and ‘Hayward’ had a similar single ‘S’ shaped growth curve (standard sigmoidal growth), which was consistent with previous reports (Burdon et al., 2017; Boldingh, Smith & Klages, 2002). Unlike ‘Hongyang’ and ‘Hayward’, the fruit weight of ‘Kuilv’ demonstrated a double ‘S’ shaped growth curve. The single ‘S’ shaped growth curve is generally consistent with the processes of cell division, cell expansion and maturation. In the double ‘S’ growth curve, initially the fruit growth is also related to cell division, as in the single S growth pattern, but later it is also affected by seed development, crop load, climate and growing conditions (Kim, Beppu & Kataoka, 2012). Moreover, the difference in growth characteristics between ‘Kuilv’ and the other two types of kiwifruit may be because ‘Kuilv’ is a cold-resistant kiwifruit, which needs to accumulate carbohydrates rapidly in the early stage and reduce fruit size to cope with severe environmental conditions such as frost in late spring (Boldingh, Smith & Klages, 2002).

The DM and TSS can reflect the taste of fruit (Boldingh, Smith & Klages, 2002). The changes of TSS were similar among the three cultivars during fruit development and increased rapidly in the last 45 days before harvest. This was consistent with other studies on DM and TSS in *A. deliciosa* (Ainalidou et al., 2016), *A. chinensis* (‘Hort16A’) (Burdon et al., 2016) and *A. arguta* (Boldingh, Smith & Klages, 2002). The rapid changes we observed may be related to the starch being converted into sugars (Ghasemnezhad, Ghorbrbanalipour & Shiri, 2013). The linear accumulation of DM in ‘Kuilv’ was different from DM accumulation patterns of *A. deliciosa*, *A. chinensis* and some other *A. arguta* cultivars (Fisk et al., 2006), which are more likely to show sigmoidal growth. The linear accumulation of DM in ‘Kuilv’ may be caused by a different accumulation mode of non-structural carbohydrates, because starch and soluble sugars comprise a significant portion of soluble solids, accounting for nearly 75% of DM (Mcglone & Kawano, 1998). The DM content of ‘Kuilv’ was the highest, even though this cultivar had the lowest fruit weight. This might be due to the higher concentration of myo-inositol, which contributes to cold tolerance of *A. arguta* (Klages et al., 1998).

Non-structural carbohydrates not only contribute to changes in DM and TSS, but are also important for flavor. The three cultivars showed similar changes in starch accumulation over the fruit development period. Before starch accumulates, there is a cell division period (Nardozza et al., 2013). Then begins the starch accumulation period, which is different among the three species. For ‘Kuilv’, starch accumulation occurred between 60 and 90 DAA, which was much shorter than that of the other two cultivars. The changes in starch during this period probably resulted from dilution caused by growth (Moscatello et al., 2011). The concentration of sugars (mainly sucrose, glucose and fructose) increased slowly over the first two stages, which is also related to an increase in water (Oh et al., 2017). After accumulation, starch is hydrolyzed into soluble sugars, and the starch content decreased until maturity in the three kiwifruit cultivars. For ‘Hayward’ and ‘Hongyang’, this was the third period, in which the hexose concentration increased drastically and the difference between sugar contents became greater. However, the accumulation of non-structural carbohydrates in ‘Kuilv’ did not change much during the third period. Sucrose contributed the most to sugar accumulation after starch hydrolysis in ‘Kuilv’, while in ‘Hayward’ and ‘Hongyang’, glucose and fructose contributed most to the sugar accumulation, consistent with previous studies (Boldingh, Smith & Klages, 2002). After harvest, the content of sucrose in ‘Kuilv’ was almost two- to three-fold of that in ‘Hayward’ and ‘Hongyang’. The high sucrose content in *A. arguta* could be explained by its relatively lower invertase activity or its increased sucrose-phosphate synthase activity compared with the other two cultivars during maturation (Mikulic-Petkovsek et al., 2012).

As reported in earlier studies, when starch starts to accumulate, citric acid levels will increase and malic acid will decrease after reaching a maximum (Ferrandino & Guidoni, 1998). While the changes in these organic acids were similar among the different cultivars, the quinic acid content varied widely (Ferrandino & Guidoni, 1998; Richardson et al., 2011; Marsh et al., 2009). In ‘Kuilv’, quinic acid continued to decrease, finally accounting for less than the other two main organic acids. In addition, the peak organic acid concentration corresponded with the maximum growth rate, due to the maintenance of osmotic pressure (Nardozza et al., 2013).

At harvest, the total organic acid content (malic + quinic + citric) is similar between *A. deliciosa* and *A. chinensis* is, while that of *A. arguta* is relatively lower (Burdon et al., 2004). Consistently, here we showed that the relatively low level of organic acid in ‘Kuilv’ ultimately resulted from the low concentrations of malic acid and quinic acid. At harvest, quinic acid in ‘Kuilv’ was almost half of that in ‘Hongyang’ and ‘Hayward’, which was related to the different quinate dehydrogenase (QDH) activities among the three species. The QDH activity is highest in *A. deliciosa* and *A. chinensis* and lowest in *A. arguta*, in parallel with the quinic acid accumulation (Marsh et al., 2009). Organic acid is also an indicator of organoleptic quality (along with sugar) (Drummond, 2013). Because quinic acid was more acidic than the other two acids (Marsh et al., 2004), and ‘Kuilv’ had a higher sugar level and lower acidity, it also had a better flavor and was expected to be more popular among consumers.

VC content is an important nutritional index of the quality of fruits and vegetables due to the roles of VC as a vitamin and an antioxidant, and in the reduction of risks of some diseases, including cancer and cerebrovascular diseases. Kiwifruits contain more VC than other VC-containing fruits such as oranges and lemons (Ma et al., 2017).

The VC content varied among fruits of the three cultivars because of their different growth characteristics. All showed a decrease of VC content at around 45 DAA, especially ‘Kuilv’, similar to the change in organic acid concentration. This period corresponded to the rapid growth period of three types of kiwifruit. The high content of VC in the early stage of rapid growth is required as a cofactor of hydroxylase, which participates in the regulation of cell growth and cell elongation of fruit (Ma et al., 2017). The growth of ‘Hongyang’ and ‘Hayward’ fruits showed a single ‘S’ curve, suggesting that there was no second period of rapid growth. However, the growth of ‘Kuilv’ showed a double ‘S’ shaped curve, suggesting a second rapid growth period. This was consistent with a previous study, which reported that VC participates in metabolism and substance biosynthesis(Ma et al., 2017). Thus, the VC content of ‘Kuilv’ decreased in the second rapid growth period, but at a much slower rate than during the first rapid growth period.

During the whole fruit development period, the VC content of ‘Kuilv’ was higher than that of ‘Hongyang’ and ‘Hayward’, and at harvest time ‘Hayward’ had the lowest VC content. These results are similar to those reported by most studies (Towantakavanit, Park & Gorinstein, 2011; Sivakumaran et al., 2016). The VC contents in *A. chinensis* ‘Golden King’ and ‘Hort16’ are higher than that in ‘Hayward’ (Towantakavanit, Park & Gorinstein, 2011; Sivakumaran et al., 2016). In addition, Leontowicz et al. (2016) found that *A. arguta* contains a high level of VC, especially in cultivars ‘M1’, ‘Geneva’ and ‘Bingo’, with the lowest content of VC found in ‘Hayward’. These studies suggested that there were differences in VC content among different cultivars and species of kiwifruit.

Plants tend to produce massive reactive oxygen species under stress, inducing a destructive impact on the growth and physiological processes of plants. Besides the nonenzymatic antioxidant system, enzymatic antioxidants such as SOD, CAT and POD can also scavenge the reactive oxygen (Xia et al., 2020; Liu et al., 2014; Liu et al., 2015). The antioxidant enzyme activities of ‘Hayward’ and ‘Hongyang’ fruits in our study were similar to those reported in previous work (Yang et al., 2015; Wang et al., 2020). However, research on the antioxidant enzyme activity of ‘Kuilv’ fruit has not been reported. Many studies have demonstrated that different antioxidant enzymes play different roles in plant stress tolerance. For example, apple enhances the activities of POD and CAT to protect leaves from oxidative stress (Mei et al., 2020). Under chilling stress, increases in SOD and POD can improve the tolerance of cucumbers to chilling temperature (Qian et al., 2012). In addition, studies have shown that genotype influences the antioxidant capacity (Cardeñosa et al., 2016; Moreno et al., 2020). In conclusion, the different antioxidant enzyme activity profiles suggested the varied stress tolerance abilities of the three kiwifruit cultivars.

## Conclusions

After comparing the growth performance and physicochemical quality of the three kiwifruit cultivars (‘Kuilv’, ‘Hongyang’ and ‘Hayward’), we found that flowering period and ripening time were dramatically different when the cultivars were planted in the same resource orchard. Furthermore, the pattern of aggregation and content of organic acids and soluble sugars were dissimilar among them. At maturity, the antioxidative enzymatic systems were also substantially different among the three kiwifruit cultivars. These findings may provide guidance in the development of different types of technologies for kiwifruit.

##  Supplemental Information

10.7717/peerj.11538/supp-1 Supplemental Information 1 Raw data: comparison of fruit morphology and nutrition metabolism in different types of kiwifruit across developmental stages

## Additional Information and Declarations

### Data Availability

The following information was supplied regarding data availability: The raw measurements are available in the Supplementary File.
